# Application of automated electron microscopy imaging and machine learning to characterise and quantify nanoparticle dispersion in aqueous media

**DOI:** 10.1111/jmi.12853

**Published:** 2019-12-18

**Authors:** M. ILETT, J. WILLS, P. REES, S. SHARMA, S. MICKLETHWAITE, A. BROWN, R. BRYDSON, N. HONDOW

**Affiliations:** ^1^ School of Chemical and Process Engineering University of Leeds Leeds U.K.; ^2^ Department of Veterinary Medicine University of Cambridge Cambridge U.K.; ^3^ Centre for Nanohealth Swansea University, College of Engineering Swansea U.K.

**Keywords:** Agglomeration, automated imaging, machine learning, nanoparticles

## Abstract

For many nanoparticle applications it is important to understand dispersion in liquids. For nanomedicinal and nanotoxicological research this is complicated by the often complex nature of the biological dispersant and ultimately this leads to severe limitations in the analysis of the nanoparticle dispersion by light scattering techniques. Here we present an alternative analysis and associated workflow which utilises electron microscopy. The need to collect large, statistically relevant datasets by imaging vacuum dried, plunge frozen aliquots of suspension was accomplished by developing an automated STEM imaging protocol implemented in an SEM fitted with a transmission detector. Automated analysis of images of agglomerates was achieved by machine learning using two free open‐source software tools: CellProfiler and ilastik. The specific results and overall workflow described enable accurate nanoparticle agglomerate analysis of particles suspended in aqueous media containing other potential confounding components such as salts, vitamins and proteins.

**Lay Description:**

In order to further advance studies in both nanomedicine and nanotoxicology, we need to continue to understand the dispersion of nanoparticles in biological fluids. These biological environments often contain a number of components such as salts, vitamins and proteins which can lead to difficulties when using traditional techniques to monitor dispersion. Here we present an alternative analysis which utilises electron microscopy. In order to use this approach statistically relevant large image datasets were collected from appropriately prepared samples of nanoparticle suspensions by implementing an automated imaging protocol. Automated analysis of these images was achieved through machine learning using two readily accessible freeware; CellProfiler and ilastik. The workflow presented enables accurate nanoparticle dispersion analysis of particles suspended in more complex biological media.

## Introduction

Nanoparticle cell uptake studies in vitro or in vivo require nanoparticles to be dispersed in biological media. Exactly how nanoparticles disperse in a particular media, whether for example as monodispersed or agglomerated species, can significantly affect factors that influence cell uptake such as size, morphology and nanoparticle dose (Chithrani *et al*., 2006; Albanese & Chan, 2011). Biological fluids are typically comprised of a number of different components, such as proteins and salts, and therefore the dispersion‐state of nanoparticles in these media is likely to differ greatly from the dispersion in simple systems such as water or even suspensions of the individual media components (Moore *et al*., 2015). As a consequence, understanding these more complex dispersion states is vital for control and prediction of nanoparticle uptake. Size‐analysis using traditional light scattering based techniques such as dynamic light scattering (DLS) is complicated, as signals deriving from the nanoparticles cannot always be separated from signals from the additional components present within biological media.

Electron microscopy (EM) can provide an alternative approach as it can directly image the dispersion at a sufficiently high spatial resolution, provided sample preparation methods are appropriately representative of the true dispersion in suspension (Brydson *et al*., 2015). Even then however, building up statistically relevant datasets is extremely time consuming when undertaken manually. Similarly data analysis via manual size measurements is time‐limiting for such large datasets, and thresholding size analysis can be difficult when artefacts from the media (salts etc.) are present in many images. To address these difficulties we present a workflow that utilises automated scanning transmission EM (STEM) imaging to collect an appropriate number of images and then employs machine learning to automate the measurement of agglomerate sizes from large image datasets. Note here our focus is on investigating nanoparticle agglomerate size rather than the primary particle size which can also be of interest (Oktay & Gurses, 2019). The use of automated imaging and analysis has been reported previously within the biological sciences (Kuwajima *et al*., 2013) for image segmentation and recently, there has been a trend towards implementing deep learning for image segmentation (Al‐Kofahi *et al*., 2018; Oktay & Gurses, 2019). However, whilst these processes have advantages, they often require considerable computer programming to implement. In comparison, in this work we aim to present a more readily accessible workflow to aid a wide range of scientific researchers.

Following validation using a simple, monodispersed model system of silica nanoparticles, we apply this workflow to analyse the dispersion of iron oxide nanoparticles in cell culture media. Iron oxide nanoparticles have shown promise in medical applications including medical imaging and drug delivery and consequently undergo numerous cell uptake screening studies (Rosen *et al*., 2012; El‐Boubbou, 2018). Using datasets collected by automated imaging and analysis of vacuum dried, plunge frozen aliquots of particle suspensions (generated following the protocol in Hondow *et al*., 2012) we show that there is a significant difference in the iron oxide agglomeration state dependent upon the exact composition of the cell culture media.

## Materials and methods

### Materials

Silica nanospheres with a primary particle diameter of 100 nm were sourced from AngstromSphere Fiber Optic centre (New Bedford, MA, USA). Iron oxide (Fe_x_O_y_) nanoparticles with tetramethylammonium hydroxide surface functionalisation and a primary particle size of 8–13 nm and aspect ratio close to 1 were sourced from the EU Horizon2020 project HISENTS, (sample code ICN_Fe_x_O_y__002) (synthesised at ICN2, Barcelona, Spain). Suspending media were Dulbecco's modified eagle medium (DMEM) cell culture media (ThermoFisher Scientific, Paisley, UK) supplemented with foetal bovine serum (FBS) (Sigma Aldrich, Poole, UK).

### Nanoparticle suspension preparation

Stock nanoparticle suspensions were prepared in water at a concentration of 1 mg mL^−1^ via ultrasonic bath sonication for 10 min. The stock suspensions were then diluted to 100 µg mL^−1^ in the appropriate media for nanoparticle dispersion characterisation. Silica nanoparticles were dispersed in deionised water; iron oxide nanoparticles were dispersed in DMEM supplemented with 0% or 10% FBS.

Sample preparation for EM imaging was carried out in two ways. For initial TEM screening of the SiO_2_ nanoparticle dispersion the sample was drop cast onto a TEM grid and left to dry in air. Second, to remove the occurrence of drying artefacts, a more representative sample preparation technique was used. A 3.5 µL drop of suspension was loaded onto a 200 mesh continuous carbon film TEM grid (EM resolutions) and rapidly plunge frozen into liquid ethane using an FEI Vitrobot©. The grid was then warmed to room temperature under vacuum using a vacuum desiccator. This vacuum sublimation process has been shown to maintain the native position of the nanoparticles in dispersion on the support film (Hondow *et al*., 2012).

### Dynamic light scattering

For bulk nanoparticle size analysis a Malvern Zetasizer Nano series ZS instrument was used to carry out dynamic light scattering (DLS). Measurements were taken directly after the sample suspensions were prepared as described above. A total of at least 3 measurements obtained from an average of 10 runs each were collected and averaged for size analysis. Refractive indices of 1.45 (Lee *et al*., 2007) and 3.00 (Lide, 2000) were used for the SiO_2_ and Fe_x_O_y_ nanoparticles respectively, and 1.33 (Hale & Querry, 1973) and 1.34 (Hoang *et al*., 2019) for water and cell culture media respectively. A temperature of 25°C was used for suspensions in water and 37°C for suspensions in media. The samples were left to equilibrate for 120 s at temperature, prior to size measurement.

### Automated imaging

**Figure 1:**
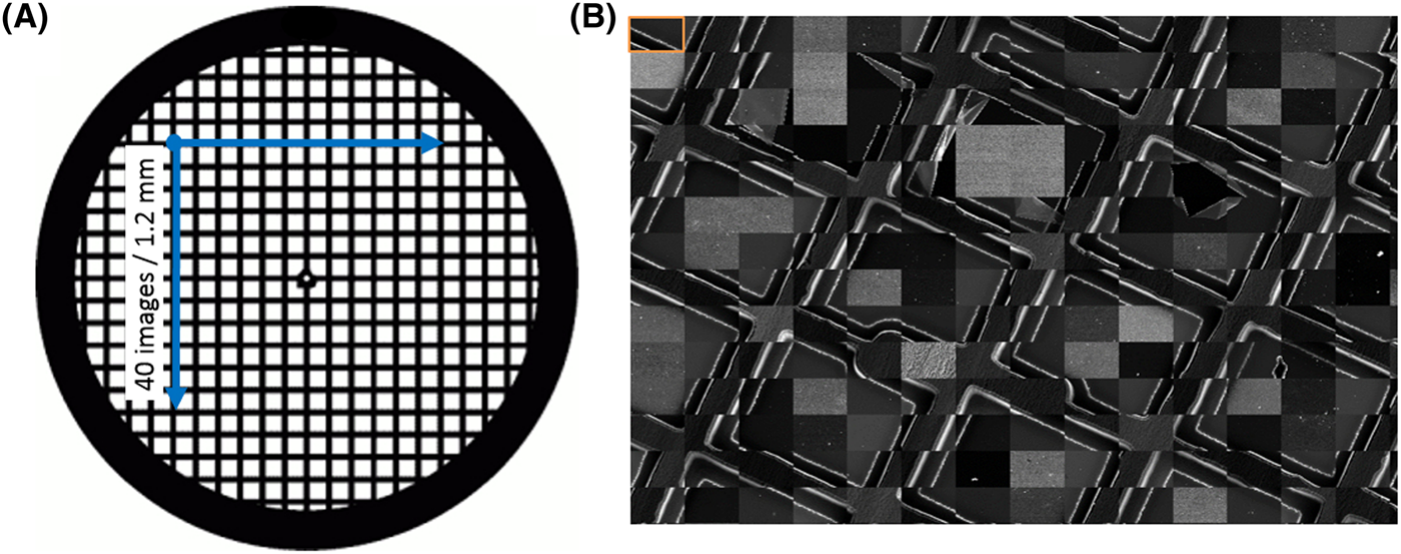
A schematic of the automated imaging workflow for a TEM grid prepared with a suspension of SiO_2_ in water by plunge freezing followed by vacuum drying. A total of 40 × 40 images were captured. (A) A large 200 mesh support grid was used to maximise the imaging area. (B) An example of part of the image grid showing 14 × 14 images stitched together. The orange box indicates the outline of one individual image.

Imaging was undertaken on an FEI Helios GA CX dualbeam Scanning EM equipped with a darkfield STEM detector. A script for automatic imaging was written using FEI's iFast developer software in order to build up statistically relevant datasets. A focusing step was applied prior to image capture (total capture time ∼50 s). Images were taken at a constant magnification row by row in a grid formation with no image overlap. For the 100 nm SiO_2_ nanoparticles 1600 images were collected in a 40 × 40 grid and each image had a horizontal field width of 30 µm. This resulted in a 1.4 mm^2^ region of the specimen being imaged (Fig. 1). For the case of smaller nanoparticles where higher magnification and thus smaller image field widths are used then multiple image grids of smaller total image number would be captured from the same specimen. The starting points of these would require initial screening of the specimen to locate reasonable areas to image. A maximum of 6 samples could be loaded in the STEM holder at any one time, and by running automated imaging over multiple days this allowed collection of large datasets. The imaging area can be maximised by using larger mesh sized TEM support grids, in this case 200 mesh.

### Data analysis – machine learning

Two readily available freeware packages were used to carry out machine analysis; CellProfiler (v 2.2.0) (Jones *et al*., 2008) and ilastik (v 1.2.3) (Berg *et al*., 2019). Following image collection, CellProfiler was used to correct the illumination of each image to remove uneven background contrast levels. Following this, a small subset of images, typically <10% of the total number of images (although the exact number depends on the size of the dataset used) were loaded into ilastik. Using the ‘pixel classification’ workflow, a ground truth was established by manually labelling pixels into one of two classes: ‘agglomerates’ or ‘background’. It should be noted that exemplifications of single particles were included in the agglomerates class, and subsequently occupy the minimum agglomerate size in the distributions shown. The live update option was used to review the predicted pixel classification after initial training and additional labels were added to the training images until accurate agglomerate identification was consistently achieved. The success of the automated strategy was then measured by comparison against the same, manually segmented fields using the Jaccard index (Jaccard, 1908) (using 2 fields‐of‐view, an agreement of 81% ± 12% was achieved across 63 agglomerate objects scored). Once the training process was completed all images from the dataset were then batch processed in order to obtain a probability image for each. This image described the probability of pixels belonging to the agglomerate or background classes. These probability images along with the original and illumination corrected images were then loaded into CellProfiler and, using a second pipeline, agglomerate objects in each of the derived probability images were identified, with size and shape measurements subsequently reported for each agglomerate‐object.

## Results and discussion

**Figure 2:**
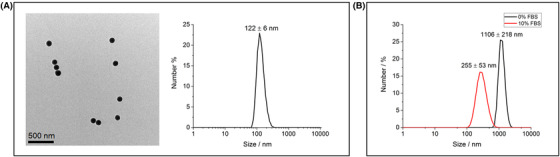
(A) TEM bright field image of a dispersion of SiO_2_ nanoparticles in water showing a primary particle size of 100 nm alongside a DLS number plot to confirm a monodisperse suspension (sample prepared for TEM by drop‐casting); (B) DLS number plots of iron oxide nanoparticles dispersed in cell culture media with and without serum protein supplementation. The primary particle size of the iron oxide nanoparticles is ∼10 nm but significant agglomeration is evident when dispersed in cell culture media without the addition of foetal bovine serum (FBS – 0%). Supplementation with 10% FBS decreases the measured agglomeration from ∼1100 nm for 0% FBS to ∼250 nm for 10% FBS.

In order to carry out statistically relevant image analysis by EM there is a requirement to collect very large datasets. Manual acquisition of such large datasets is not possible in a time efficient manner and can also be subject to user bias. Consequently, there is a need to automate the imaging process as well as the analysis. A model, monodispersion of silica nanoparticles in water was identified by dynamic light scattering and TEM (Fig. 2A) and used to design and establish an automated STEM in SEM imaging script in iFast developer to provide a platform to collect datasets in excess of 1000 images per sample automatically. Collection of such large image datasets brings the need to automate an appropriate quantitative image data analysis procedure. This was achieved using the images obtained from the model, monodispersed, silica nanoparticle sample and resulted in a workflow using the open source ilastik and CellProfiler softwares, as described in the methods section. Once initial machine training using ilastik pixel classification was accomplished, image segmentation and analysis of >500 images could be achieved within ∼10 min (although this will vary depending on the specifications of the computer used).

To take this work further a complex system of iron oxide nanoparticles dispersed in cell culture media with and without serum supplementation was then investigated. This is highly relevant to a large field of research looking at the application of superparamagnetic iron oxide nanoparticles (SPIONs) in nanomedicine where often SPIONs are required to be dispersed in a variety of cell culture media for cell uptake studies (Singh *et al*., 2012). Two iron oxide dispersions were used which exhibited significant differences in agglomeration state by dynamic light scattering (Fig. 2B). Thus providing an initial test of both the reliability of the proposed workflow and its applicability to more complex systems than monodispersion of nanoparticles in water.

### Validation

**Figure 3:**
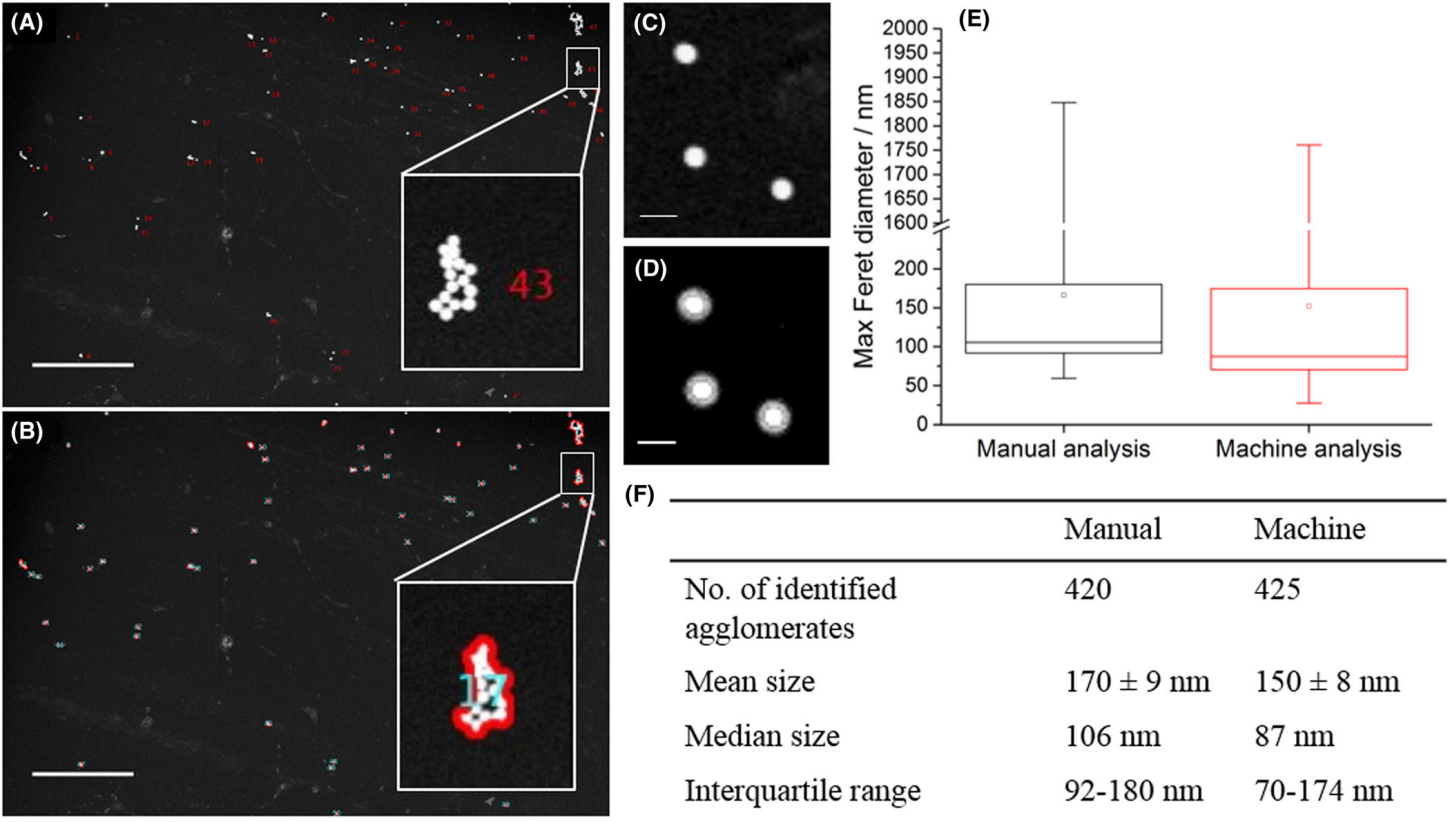
(A) Dark field STEM image of silica nanoparticles dispersed in water with manual identification of each nanoparticle agglomerate. This was compared to the machine segmentation of the same STEM image (B). The insets in both STEM images show an enlarged region (white box) indicating more clearly a specific nanoparticle agglomerate. The white scale bar indicates a distance of 5 µm. (D) Example of the focal halo that can erroneously be included in image segmentation in an exported probability image from ilastik; (C) shows the same area of the original, image (the white scale bars represent a distance of 200 nm). (E) A comparison between the Feret diameter measured manually and by machine analysis is shown using a box and whisker plot, presenting the interquartile range (the box), the median (‐) the mean (▫) and the overall range of the data. There was no significant difference between the two datasets (*p* > 0.05). (F) A summary of the measurement data of the Feret diameters shown in the box and whisker plot indicating that there was good agreement between the manual and machine learning approaches. The standard error of the mean is reported for uncertainty values.

**Figure 4:**
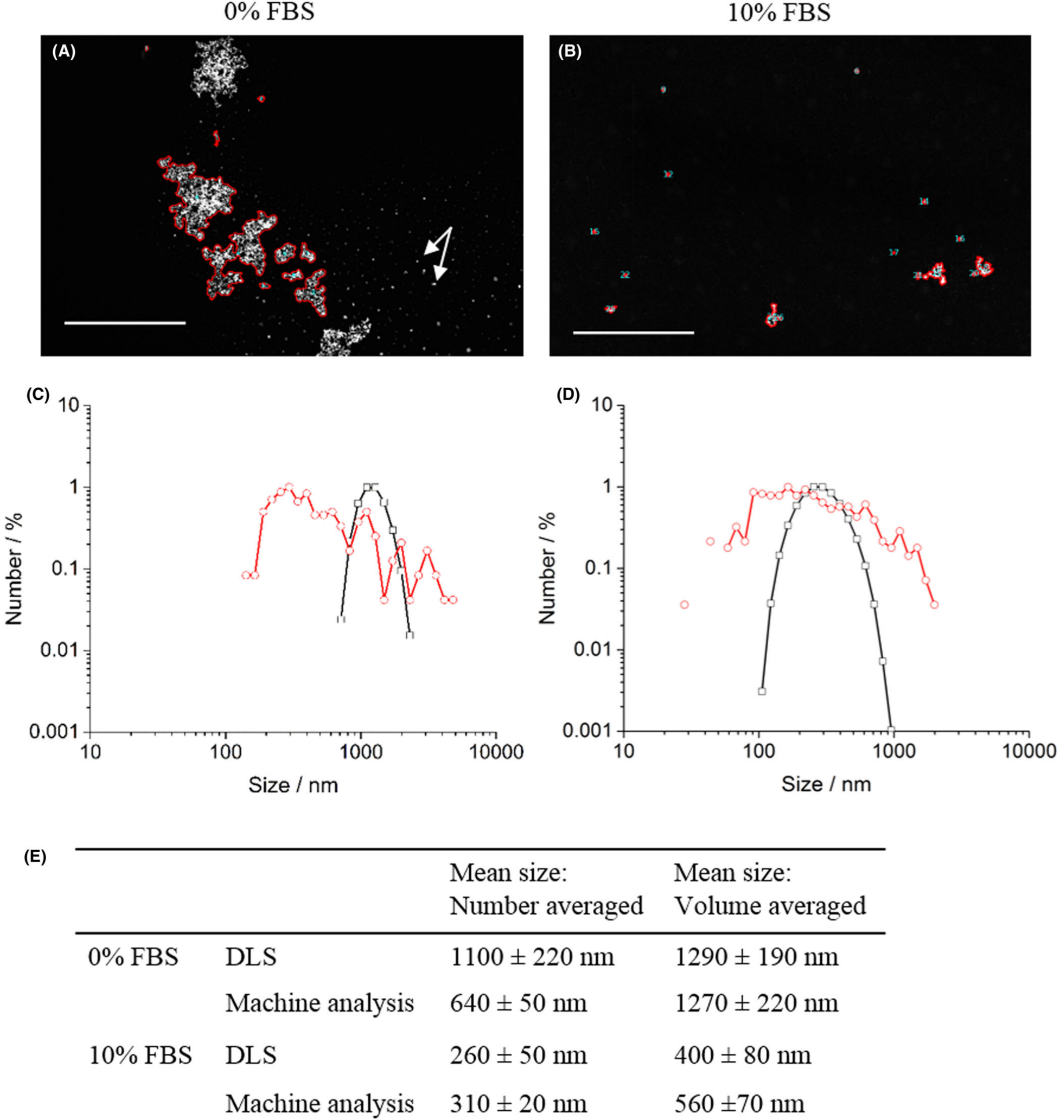
Dark field STEM images from TEM grids prepared from iron oxide nanoparticles dispersed in cell culture media with 0% (A) and 10% (B) FBS. Successful segmentation of nanoparticles from salts (indicated by the white arrows in (A)) was achieved and the white scale bar indicates 5 µm. Number distributions of agglomerate size for EM data analysis by machine learning (red) and for DLS analysis (black) are shown for both systems; 0% FBS (C) and 10% FBS (D). Table (E) presents the mean values from DLS and EM data analysis for both samples calculated using both a number and volume distribution. A larger degree of agglomeration with complex shapes was seen in the 0% FBS suspension. Good agreement between EM and DLS analysis was seen for the 10% FBS sample, but there was some discrepancy in the 0% FBS sample that may be attributed to overweighting of larger agglomerates in DLS scattering analysis. This was reduced when volume averaged diameters were compared.

To ensure the proposed segmentation and analysis was accurate, the workflow was validated via comparison with a manual analysis. 10 dark field STEM images of the monodispersed silica nanoparticles plunge frozen and then vacuum dried to retain the native position of the nanoparticles in water were used for manual segmentation to identify each nanoparticle or nanoparticle agglomerate within the images, with a total of 420 objects identified (Fig. 3). In comparison 425 objects were identified when segmentation of the same 10 images was carried out by machine learning analysis. Thus the identification of agglomerates was comparable (Figs. 3A, B). The slight discrepancy arises from artefacts, most likely due to small contaminants within the sample suspension and damage to the grid during sample preparation being erroneously identified as agglomerates by machine analysis. However the 1% difference in the identification of the agglomerates by machine learning analysis was deemed to be acceptable. Whereas we note that here, simple thresholding and particle counting may have been successful for agglomerate segmentation and measurement, such an approach cannot address agglomerate measurements in more complex systems. For example when using cell culture media, salts can precipitate onto the TEM grid during sample preparation. In this case segmentation by simple thresholding and particle counting would fail to separate salts from nanoparticles, whereas segmentation by using machine learning is able to ‘recognise’ one from the other; correctly assigning salts to the background class (Fig. 4A).

Second, size measurements of the STEM images of the dispersed silica nanoparticles were carried out manually in Gatan Microscopy Suite (v 3.0.1) by measuring the Feret diameter of each agglomerate. These measures were compared to the exported size measurements obtained by automated analysis using CellProfiler. Initially we found that measurements by machine analysis were consistently overestimated resulting in a mean size of nanoparticle agglomerates of 210 ± 8 nm as compared to 170 ± 9 nm obtained by manual size analysis. This may be an imaging artefact due to small focal variations since in trying to maximise the image area by utilising a low magnification, small contrast differences due to focus can extend beyond the outer pixel edge of the nanoparticle agglomerates causing a halo around some of the particles (Figs. 3C, D). With manual analysis this can be easily identified and compensated for and is thus not included in the agglomerate measurements. To reduce the impact in the automatically acquired data, an object erosion step was added to the pipeline in CellProfiler by introducing the ExpandorShrinkObject analysis module which was set to erode each identified object by 1 pixel. This resulted in better agreement between the manual and machine learning measurements when comparing the mean, median and interquartile range of the measured Feret diameter (Fig. 3E). The analysis showed a largely monodisperse system, where the average agglomerate size was comparable to the primary particle size (100 nm), as would be expected from the DLS results (Fig. 2A). Additionally a two‐sample unpaired *t*‐test was carried out using OriginPro v2016 (Origin(Pro) Version 2016) and showed there was no significant difference between the two datasets (*p* > 0.05).

### Application: Iron oxide nanoparticles in cell culture media

Iron oxide nanoparticles were dispersed in cell culture media with and without the addition of a common protein supplement, FBS and then prepared for STEM by vacuum drying, blotted and plunge frozen aliquots of particle suspensions. A significant difference between the agglomeration state of iron oxide nanoparticles was observed between the two media compositions by STEM, with far larger agglomeration being observed when no FBS was present in the cell culture media (Fig. 4). This is broadly consistent with the results gained by DLS (Fig. 2B). We note however that whilst the DLS results agree well with the EM data for the 10% FBS system where the mean diameter was measured as 310 ± 20 nm and 260 ± 50 nm from EM data analysis and DLS analysis respectively (Fig. 4D), there is a degree of discrepancy for the 0% FBS samples where the measured mean diameter was smaller from EM data analysis (640 ± 50 nm) compared to DLS analysis (1100 ± 210 nm) (Fig. 4C). We attribute this discrepancy to the difference in how the size distributions are measured by the two techniques. For EM data analysis every particle is counted individually whilst DLS measures scattering intensity. Since light scattering is proportional to *d* ^6^ where d is the diameter of a particle, larger particles tend to dominate in DLS analysis (Gebhart, 1991). Accordingly we found that the average diameter from the EM data increased to 1270 ± 220 nm for the 0% FBS system if a volume rather than number average diameter was calculated which is in closer agreement to the DLS data. The number averaged diameter was simply the average of the Feret diameter of each measured agglomerate. In comparison the volume averaged diameter was obtained by estimating the volume of each agglomerate (from the Feret diameter), averaging to obtain the mean agglomerate volume, and then estimating the volume averaged diameter from this value. Perhaps more importantly, the EM is clearly counting more small particle agglomerates and this may be significant if the finest particle fraction is deemed to be more active in the system of interest. An additional advantage of using EM imaging and analysis over DLS is that information regarding agglomerate shape can also be obtained; in fact the described workflow allows up to 20 different size and shape attributes of the nanoparticle agglomerates to be measured. These include for example: ‘form factor’, a measure of the circularity of an agglomerate; the major and minor axis lengths which can be valuable for analysing noncircular objects; and also perimeter measurements which can provide surface area information which has been suggested to be linked with nanoparticle toxicity (Oberdörster *et al*., 2005). These additional measurements are important since nanoparticle agglomerate size *and* shape are known to effect bionano interactions (García‐Álvarez *et al*., 2018) which can subsequently influence cellular uptake studies (Huang *et al*., 2010; Hoshyar *et al*., 2016). Care however, must be taken to account for the impact of the pre‐freezing blotting process on the projected shape of agglomerates (Wills *et al*., 2017). Appropriately corrected shape information is very difficult to obtain by manual analysis or indeed by DLS where a spherical shape is always assumed, thus demonstrating further promise of the proposed workflow.

The difference in agglomeration state of the iron oxide nanoparticles due to the presence of the serum proteins in FBS can be explained by a protein interaction with the surface of nanoparticles, surrounding them with a layer known as the protein corona (Cedervall *et al*., 2007; Treuel *et al*., 2014). This corona can stabilise particle dispersions by screening the effect of the high ion concentration in cell culture media and as a consequence can reduce agglomeration, as was observed here (Allouni *et al*., 2009; Wang *et al*., 2019).

In this work we have applied an automated imaging and analysis workflow to two relatively simple nanoparticle dispersions. In using a preexisting simple machine learning software that utilises sparse labelling, we avoided complications associated with other machine and deep learning approaches which require a large amount of more precise masks of the objects as training data in order to be accurate. We believe the approach can be applied to more complex systems that require detailed image segmentation and/or size analysis. Such examples could include: the analysis of different nanoparticles dispersed in a range of biological environments and; the identification and quantification of nanoparticles within resin embedded, cellular thin‐sections produced from cell uptake studies. We have previously applied semi‐automated analysis methods to both these types of datasets (Hondow *et al*., 2012; Summers *et al*., 2013), however by the implementation of a largely automated workflow, including significantly, both data acquisition and analysis, there would be a rapid increase in the speed at which statistically relevant amounts of data could be generated. To achieve this, continued development of the automated imaging set‐up will be required. Thus far, we have found that out of focus image acquisition occurred when no obvious features were in the field of view. This can be overcome by using lower magnifications potentially limiting the size of the particles that can be measured, or using a higher concentration of nanoparticles on the grid. However, there are opportunities to explore automated imaging within TEM through software options such as FEI's MAPS image acquisition which could address some of these issues. In addition initial attempts to automate imaging of resin embedded, cellular thin‐sections indicate further considerations regarding electron beam damage to less stable samples will be required. However, notwithstanding these difficulties, we believe datasets of 500 usable images should be consistently achievable, the analysis of which would be realisable within rapid time scales of hours. Furthermore, using readily available freeware which is extremely user friendly, particularly for researchers without prior knowledge of machine learning analysis, ensures that the proposed workflow is widely accessible.

## Conclusion

In summary, we present and validate an automated agglomerate measurement approach using machine learning and demonstrate it to be a promising technique for the characterisation of the dispersion state of nanoparticles in biological fluids. Automated STEM imaging has been utilised to build up statistically relevant amounts of image data and has been coupled with machine learning analysis; both processes significantly reduce the time required to carry out an accurate analysis via electron microscopy. Finally, the proposed workflow has been used to confirm that iron oxide nanoparticles agglomerate in cell culture media without the presence of surface‐stabilising serum proteins, additionally revealing the complexity of the agglomerate morphologies in the absence of serum proteins.
